# Comparison of self-reported and register-based hospital medical data on comorbidities in women

**DOI:** 10.1038/s41598-019-40072-0

**Published:** 2019-03-05

**Authors:** Peh Joo Ho, Chuen Seng Tan, Shajedur Rahman Shawon, Mikael Eriksson, Li Yan Lim, Hui Miao, Eileen Png, Kee Seng Chia, Mikael Hartman, Jonas F. Ludvigsson, Kamila Czene, Per Hall, Jingmei Li

**Affiliations:** 1 Genome Institute of Singapore, 60 Biopolis Street, Genome, #02-01, Singapore, 138672 Singapore; 2 Saw Swee Hock School of Public Health, National University of Singapore and National University Health System, Singapore, Singapore; 3 Cancer Epidemiology Unit, Nuffield Department of Population Health, University of Oxford, Old road campus, OX3 7LF Oxford, UK; 4 Karolinska Institutet, Department of Medical Epidemiology and Biostatistics, Box 281, 171 77 Stockholm, Sweden; 5 Department of Surgery, University Surgical Cluster, National University Hospital, Singapore, Singapore; 6 Department of Surgery, Yong Loo Lin School of Medicine, National University of Singapore, Singapore, Singapore; 7 Department of Pediatrics, Örebro University Hospital, Örebro University, Örebro, Sweden; 8 Department of Oncology, Södersjukhuset, 118 84, Stockholm, Sweden

## Abstract

Breast cancer patients commonly present with comorbidities which are known to influence treatment decisions and survival. We aim to examine agreement between self-reported and register-based medical records (National Patient Register [NPR]). Ascertainment of nine conditions, using individually-linked data from 64,961 women enrolled in the Swedish KARolinska MAmmography Project for Risk Prediction of Breast Cancer (KARMA) study. Agreement was assessed using observed proportion of agreement (overall agreement), expected proportion of agreement, and Cohen’s Kappa statistic. Two-stage logistic regression models taking into account chance agreement were used to identify potential predictors of overall agreement. High levels of overall agreement (i.e. ≥86.6%) were observed for all conditions. Substantial agreement (Cohen’s Kappa) was observed for myocardial infarction (0.74), diabetes (0.71) and stroke (0.64) between self-reported and NPR data. Moderate agreement was observed for preeclampsia (0.51) and hypertension (0.46). Fair agreement was observed for heart failure (0.40) and polycystic ovaries or ovarian cysts (0.27). For hyperlipidemia (0.14) and angina (0.10), slight agreement was observed. In most subgroups we observed negative specific agreement of >90%. There is no clear reference data source for ascertainment of conditions. Negative specific agreement between NPR and self-reported data is consistently high across all conditions.

## Introduction

Cancers including breast cancer commonly present with one or more additional medical conditions, hereafter referred to as comorbidities. Comorbidities are known to influence treatment decisions and ultimately survival of breast or other cancers^1,2^. In breast cancer patients, presence of comorbidities was found to increase the likelihood of being diagnosed with advanced disease, for example, with distant metastasis^3^. These patients were more likely to be treated with less-than-standard therapy than patients with no comorbidities^4,5^. In addition, delay and non-completion of adjuvant therapies were more frequent in this group of patients with comorbidities^6^. The risk of dying from breast cancer was also higher among breast cancer cases with a history of diabetes or myocardial infarction^1^.

Information on comorbidities can be ascertained through multiple data sources including patient self-reports, medical record abstraction and disease registries^7^. National registers such as the National Patient Register (NPR) in Sweden are goldmines for epidemiological research due to their rich and long-term data on various health conditions and procedures^8^. The NPR was established in 1964, and achieved nationwide coverage for all in-patient visits in 1987^9^. It contains detailed information about the patient, geographical data, administrative data for both inpatient and outpatient visits and codes for related medical diagnosis and procedures. The register, however, does not yet contain primary care data. A validation study by Ludvigsson *et al*. using only inpatient records showed high positive predictive value (i.e. the probability of truly having the condition given that the inpatient records reports it) (85–95% in general) but lower sensitivity (i.e. the probability of inpatient records reporting the condition given that the condition is present) for many diagnoses in the NPR^9^.

Comorbid conditions such as hypertension and adulthood onset diabetes are almost exclusively managed in primary care and are therefore not well-captured in the register-based hospital records^10^. Ludvigsson *et al*. noted that sensitivity captured by inpatient records were especially low for hypertension and lipid disorders in the Swedish NPR (~10%)^9^. Other studies have highlighted the under-recording of mild medical conditions that do not require hospital care in hospital admission data^11,12^. Therefore, self-reported data from the patients may bridge the gap in recording these conditions and be an important source of information^13^. Though the reliability of self-reported medical conditions varies considerably, it has been increasingly adopted in both research and clinical settings^13^. The accuracy of self-reported medical history is impacted by the individual’s age, health status and formal education, with higher accuracy linked to younger age, better health and higher education^14–17^. Potentially due to increased monitoring of heart disease and related conditions, higher body mass index (BMI) may also be associated with higher accuracy of self-reported medical history^18,19^.

In practice, missed cases, under- and over-reporting are inevitable regardless of data source used. However, the key question remains as to how and under what conditions different data resources can be used for research. Detailed knowledge of the limitations of different data sources can help to critically interpret results and draw conclusions from large-scale population-based research and clinical studies. Therefore, the aim of this study is to compare self-reported and register-based hospital medical data in the Swedish NPR on comorbidities in a large breast cancer screening cohort of women in Sweden. We focused on common comorbidities such as hypertension, hyperlipidemia, heart failure, myocardial infraction, angina, stroke and type I or II diabetes. In addition, we examined concordance between self-reported and NPR data on several women’s health problems which are less studied, such as preeclampsia and polycystic ovaries or ovarian cysts.

## Methods

### Study population

The KARolinska MAmmography Project for Risk Prediction of Breast Cancer (KARMA) study (http://karmastudy.org/) was set up to be a well-characterized breast cancer cohort^20^. Participants of the prospective KARMA study comprise women attending mammography screening or clinical mammography at four hospitals in Sweden (Stockholm South General Hospital, Helsingborg Hospital, Skåne University Hospital, Lund, and Landskrona Hospital). Since 1994, all women in Sweden, aged 40–74 years, are invited for publicly-funded mammography screening every 18–24 months. Adherence to mammography screening is high – three in four eligible women attend screening regularly. All women who were invited for screening between January 2011 and March 2013, at the four hospitals were invited to participate in the KARMA study. Additionally, women who had a clinical mammography (i.e. woman being referred for a mammogram because of symptom noticed by her and/or her doctor) at any of the participating mammography units during the recruitment period were invited. Of 210,233 women who were invited to participate in the KARMA study, 70,877 (34%) were enrolled. These women answered a detailed web questionnaire (https://karmastudy.org/wp-content/uploads/2015/07/Karma_baseline_questionnaire_eng.pdf) on background and lifestyle risk factors. Consent was obtained for the retrieval of data from medical records and national registers. Ninety-two percent (*n* = 65,231) of the enrolled women completed the web questionnaire. The ethical review board in Stockholm approved the study (2010/958–31/1) and all study procedures were performed in accordance with relevant guidelines and regulations.

### Data sources of medical history

In KARMA, self-reported data was collected for the following conditions: high blood pressure (hypertension), high blood cholesterol (hyperlipidemia), myocardial infarction, angina, heart failure, stroke, polycystic ovaries or ovarian cysts, preelampsia, and diabetes. Given instructions to “choose all that apply”, the participants were asked - *“Have you ever been diagnosed with [any of the medical conditions] by a medical doctor?”* For each condition, participants would mark the corresponding checkbox (i.e. yes) if they have ever been diagnosed with the specified conditions, unmarked checkbox corresponds to not having the condition (i.e. no). Women who responded *“Don’t know/Refuse”* to the question were excluded from further analysis (*n* = 270).

All participating women in KARMA study were electronically linked to the NPR (linkage date 1^st^ October 2013) through unique personal identity numbers (i.e. personnummer)^21^. Inpatient/outpatient diagnoses (main and secondary) of the nine medical conditions studied were identified using International Classification of Diseases (ICD) diagnosis codes (see Supplementary Table 1). Diagnoses registered after the date of completion of questionnaire were excluded.

### Other covariates

Self-reported information on age at time of survey, education level, BMI and smoking were derived from the KARMA web questionnaire.

### Statistical analysis

The count and percent of diseases for each data source (i.e. self-reported, and NPR) and various combinations of each medical history status combination from the two data sources (i.e., “Self-reported No/ NPR No”, “Self-reported Yes/ NPR No”, “Self-reported No/ NPR Yes” and “Self-reported Yes/ NPR Yes”) were computed. In the comparison of self-reported and NPR we report the difference in prevalence instead of percentage difference. This was chosen to avoid the impression of either source is the gold standard. Both methods gave similar results. The “epi.kappa” function in the “epiR” package was used to compute observed proportion of agreement (overall agreement), expected proportion of agreement, prevalence index, bias index, prevalence and bias corrected kappa statistic and Cohen’s Kappa statistic in R (version 3.4.2). The prevalence index ($\frac{[{\rm{y}}\,/\,{\rm{y}}]-[{\rm{n}}\,/\,{\rm{n}}]}{N}$, from the cells of a standard 2 × 2 matrix, Supplementary Method), an estimate of the difference in the probability of the condition being present and absent from the study population, ranges from −1 to 1 and equates to 0 when 50% of the study population has the condition. A larger absolute prevalence index value results in larger chance agreement and smaller Kappa value^22^. A bias index ($\frac{[{\rm{y}}\,/\,{\rm{n}}]-[{\rm{n}}\,/\,{\rm{y}}]}{N}$), the difference in the reported proportion of the condition being present between NPR and self-reported, ranges from −1 to 1 and large absolute values indicate bias increasing *κ*, whereas zero bias index indicates equal marginal proportions and no bias^22^. Kappa coefficients have the following interpretations: ≤0: no agreement; 0.01–0.20: slight agreement; 0.21–0.40: fair agreement; 0.41–0.60: moderate agreement; 0.61–0.80: substantial agreement; 0.81–1.00: almost perfect agreement^23^. Proportion of positive specific agreement, the proportion that tests positive under both criteria compared with the average proportion that test positive under each criteria separately ($\frac{2\times [{\rm{y}}\,/\,{\rm{y}}]}{N+[{\rm{y}}\,/\,{\rm{y}}]-[{\rm{n}}\,/\,{\rm{n}}]}$) was expressed as percentage (100 denotes perfect agreement)^24^. The positive specific agreement is the inverse transformed mean of the sensitivity and positive predictive values, i.e., sensitivity agnostic to which measure is the gold standard (Supplementary Method). Similarly negative specific agreement, the proportion that tests negative under both criteria compared with the average proportion that test negative under each criteria separately ($\frac{2\times [{\rm{n}}\,/\,{\rm{n}}]}{N-[y\,/\,y]+[{\rm{n}}\,/\,{\rm{n}}]}$) was expressed as percentage.

Two-stage logistic regression models for analysing agreement which takes into account chance agreement were used to identify potential predictors of overall agreement (i.e. “Self-reported No/ NPR No”, and “Self-reported Yes/NPR Yes” were coded as “1”, otherwise coded as “0”)^25^. The offset term was determined by the variable(s) in the main model. The 95% confidence interval was obtained using the bootstrap approach, the 2.5 and 97.5 percentile of 2000 iterations. The following predictors were considered: age, education level, BMI, and reported ever smoked for one year or 100 cigarettes (smoking). Stratified analyses were carried out for each condition by age (<50, 50–59 or ≥60 years), education level (elementary, intermediate or university), BMI (<25 or ≥25 kg/m^2^), and smoking (Yes or No). A subset of parous women (i.e. who had at least one full-term pregnancy) was used to study preeclampsia. Statistical significance threshold was set at P < 0.05.

### Ethics Approval And Consent To Participate

All participants signed informed consent forms, and the ethical review board at Karolinska Institutet approved the study (2010/958-31/1). All study procedures were performed in accordance with relevant guidelines and regulations.

## Results

A total of 64,961 women, aged between 21 to 87 years [mean (SD): 54.8 (10.0) years] were analysed. Descriptive statistics of participants are given in Table 1. Approximately half of the participants completed university education (*n* = 29,400; 45.3%), reported a BMI below 25 kg/m^2^ (*n* = 35,700, 55.0%) and smoking status yes (*n* = 34,274, 52.8%) in the questionnaire.

**Table 1:** Characteristics of 64,961 women attending mammography units in the KARMA study.

Characteristics			
Total number of women, *n* (%)	64,961 (100.0)		
Age at recruitment in years, mean (SD)	54.8 (10.0)		
Education level, *n* (%)			
Elementary	7,793 (12.0)		
Intermediate	17,721 (27.3)		
University	29,400 (45.3)		
Self-reported BMI at recruitment, *n* (%)			
<25 kg/m^2^	35,700 (55.0)		
≥25 kg/m^2^	28,800 (44.3)		
Smoking, *n* (%)			
Yes, *n* (%)	34,274 (52.8)		
No, *n* (%)	30,380 (46.8)		
**Prevalence of medical conditions**, ***n*** **(%)**	**Self-reported**	**NPR**	**Difference in prevalence (Self-reported – NPR)**
Hypertension	12,880 (19.8)	5,582 (8.6)	11.2
Hyperlipidemia	7,033 (10.8)	819 (1.3)	9.6
Heart failure	373 (0.6)	287 (0.4)	0.1*
Myocardial infarction	482 (0.7)	426 (0.7)	0.1*
Angina	585 (0.9)	7,611 (11.7)	−10.8
Stroke	696 (1.1)	942 (1.5)	−0.4
Polycystic ovaries or ovarian cysts	5,980 (9.2)	3,317 (5.1)	4.1
Preeclampsia^a^	2,930 (5.0)	3,456 (5.9)	−0.9
Diabetes	1,719 (2.6)	1,380 (2.1)	0.5

BMI: Body mass index; NPR: National patient register; SD: Standard deviation.

^a^Includes women who had at least 1 pregnancy (*n* = 58,936).

*Discrepancy due to rounding errors.

According to the self-reported data, the five most commonly diagnosed conditions were hypertension (19.8%), hyperlipidemia (10.8%), polycystic ovaries or ovarian cysts (9.2%), preeclampsia (5.0%) and diabetes (2.8%) (Table 1). The remaining conditions (i.e. heart failure, myocardial infarction, angina and stroke) each affected ~1% of the study population. When comparing self-reported data to the NPR, the largest differences were observed for hypertension (11.2% more in self-reported data), angina (10.8% less in self-reported data), hyperlipidemia (9.6% more in self-reported data) and polycystic ovaries or ovarian cysts (4.1% more in self-reported data) (Table 1). Differences between self-reported and NPR data were minimal (<1.0%) for heart failure, myocardial infarction, stroke, preeclampsia and diabetes.

**Figure 1:**
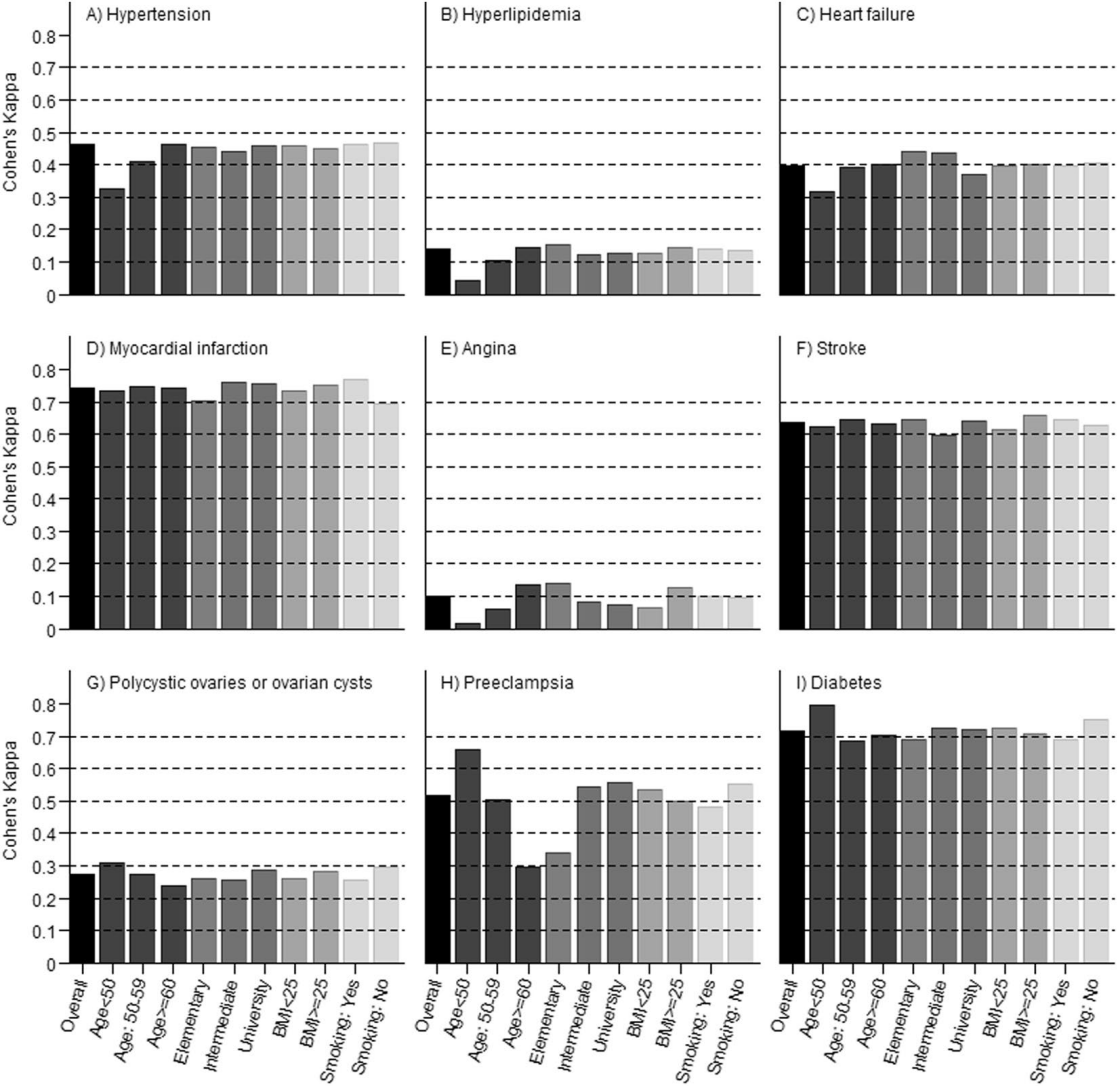
Cohen’s Kappa for commonly diagnosed conditions.

**Figure 2:**
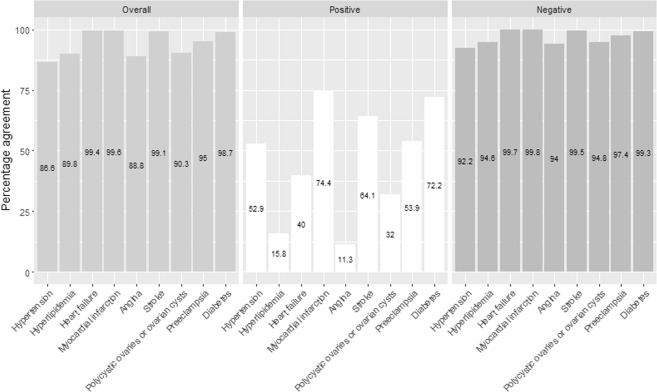
Percentage overall, positive and negative specific agreement between self-reported and register-based hospital medical data.

Figure 1 shows estimates of Cohen’s Kappa for commonly diagnosed conditions. Relative cell counts, expected proportion of agreement, prevalence index, bias index, and Cohen’s Kappa statistic are presented in Supplementary Tables 2–6. Substantial agreement (Cohen’s Kappa) was observed for myocardial infarction (0.74), diabetes (0.71) and stroke (0.64). Moderate agreement was observed for preeclampsia (0.51) and hypertension (0.46). Fair agreement was observed for heart failure (0.40) and polycystic ovaries or ovarian cysts (0.27). For hyperlipidemia (0.14) and angina (0.10), slight agreement was observed between self-reported and NPR data. High levels of overall agreement (i.e. 86.6% or more) were observed for all included conditions (Fig. 2). The average agreement between self-reported and NPR data on absence of medical condition (percent negative specific agreement, range: 92.2–99.8%) was higher than for presence (percent positive specific agreement, range: 11.3–74.4) (Fig. 2).

In multivariate two-stage logistic regression analysis (OR [95% CI]), with age, education, BMI, and smoking status in the models, older age (age ≥60 vs age <50: 1.54 [1.46–1.62]) and higher BMI (≥25 vs <25: 1.06 [1.02–1.10]) were associated with higher agreement for hypertension (Table 2). Similarly, older age (age ≥60 vs age <50: 1.15 [1.12–1.17]) and higher BMI (≥25 vs <25: 1.07 [1.04–1.09]) were associated with higher agreement for angina (Table 2). Older age (age ≥60 vs age <50: 1.15 [1.11–1.19]) was associated with higher agreement for hyperlipidemia (Table 2). Older age (age ≥60 vs age <50: 0.74 [0.60–0.92]) was associated with lower agreement for diabetes (Table 2). No smoking was associated with higher agreement for diabetes (no vs yes: 1.23 [1.07–1.43]) but lower agreement for myocardial infarction (no vs yes: 0.75 [0.57–0.98]) (Table 2). Age, education, BMI, and smoking status were not associated with agreement for heart failure and stroke. For polycystic ovaries or ovarian cysts, older age (age ≥60 vs age <50: 0.89 [0.85–0.93]) was associated with lower agreement, and higher BMI (≥25 vs <25: 1.05 [1.01–1.09]) and no smoking (no vs yes: 1.04 [1.00–1.09]) was associated with higher agreement (Table 2). In the subset of parous women, older age (age ≥60 vs age <50: 0.48 [0.44–0.52]) was associated with lower agreement, and higher education (university vs elementary: 1.15 [1.04–1.28]) and no smoking (no vs yes: 1.09 [1.01–1.17]) were associated with higher agreement for preeclampsia (Table 2).

**Table 2:** Odds ratio and corresponding 95% confidence intervals for overall agreement in each medical condition. Significant associations (P < 0.05) are denoted in bold.

Condition	Age 50–59 (*n* = 18,797) vs Age <50 (*n* = 23,154)	Age ≥60 (*n* = 23,010) vs Age <50 (*n* = 23,154)	Intermediate (*n* = 17,721) vs Elementary (*n* = 7,793)	University (*n* = 29,400) vs Elementary (*n* = 7,793)	BMI ≥ 25 (*n* = 28,800) vs BMI < 25 (*n* = 35,700)	Smoking No (*n* = 30,380) vs Smoking Yes (*n* = 34,274)
*Unadjusted*						
Hypertension	**1.24 (1.18–1.31)**	**1.59 (1.51–1.67)**	**0.86 (0.80–0.91)**	**0.87 (0.82–0.92)**	**1.11 (1.07–1.15)**	0.98 (0.94–1.02)
Hyperlipidemia	**1.08 (1.04–1.11)**	**1.16 (1.13–1.20)**	**0.94 (0.90–0.98)**	**0.95 (0.91–0.98)**	**1.03 (1.00–1.06)**	0.99 (0.97–1.02)
Heart failure	1.12 (0.80–1.51)	1.15 (0.85–1.49)	0.98 (0.76–1.25)	0.88 (0.71–1.09)	1.01 (0.86–1.19)	1.01 (0.87–1.20)
Myocardial infarction	1.05 (0.59–1.80)	1.05 (0.62–1.65)	1.24 (0.88–1.79)	1.20 (0.86–1.68)	1.07 (0.81–1.37)	**0.75 (0.57–0.98)**
Angina	**1.05 (1.03–1.07)**	**1.17 (1.15–1.19)**	**0.92 (0.88–0.95)**	**0.91 (0.87–0.94)**	**1.08 (1.06–1.10)**	0.99 (0.97–1.01)
Stroke	1.07 (0.81–1.42)	1.06 (0.82–1.35)	0.87 (0.70–1.08)	0.97 (0.79–1.19)	1.13 (0.96–1.33)	0.95 (0.81–1.10)
Polycystic ovaries or ovarian cysts	**0.95 (0.90–1.00)**	**0.89 (0.85–0.94)**	1.00 (0.93–1.06)	1.04 (0.97–1.10)	1.04 (1.00–1.08)	**1.06 (1.01–1.10)**
Preeclampsia^a^	**0.67 (0.62–0.73)**	**0.45 (0.42–0.49)**	**1.50 (1.35–1.67)**	**1.53 (1.39–1.68)**	0.95 (0.88–1.01)	**1.18 (1.10–1.27)**
Diabetes	**0.66 (0.52–0.83)**	**0.71 (0.58–0.87)**	1.09 (0.90–1.32)	1.07 (0.89–1.27)	0.98 (0.84–1.14)	**1.26 (1.09–1.45)**
Multivariable-adjusted, all covariates shown						
Hypertension	**1.24 (1.17–1.31)**	**1.54 (1.46–1.62)**	0.99 (0.93–1.06)	1.00 (0.94–1.06)	**1.06 (1.02–1.10)**	1.01 (0.97–1.05)
Hyperlipidemia	**1.07 (1.04–1.11)**	**1.15 (1.11–1.19)**	0.97 (0.93–1.01)	0.97 (0.94–1.01)	1.02 (0.99–1.05)	1.00 (0.97–1.03)
Heart failure	1.13 (0.80–1.52)	1.16 (0.85–1.50)	1.00 (0.77–1.29)	0.89 (0.71–1.12)	1.00 (0.84–1.18)	1.02 (0.87–1.22)
Myocardial infarction	1.02 (0.57–1.75)	1.07 (0.63–1.70)	1.26 (0.88–1.84)	1.24 (0.88–1.77)	1.08 (0.82–1.39)	**0.75 (0.57–0.98)**
Angina	**1.04 (1.03–1.07)**	**1.15 (1.12–1.17)**	0.97 (0.93–1.01)	**0.96 (0.92–0.99)**	**1.07 (1.04–1.09)**	1.01 (0.99–1.03)
Stroke	1.05 (0.79–1.39)	1.00 (0.77–1.28)	0.87 (0.69–1.08)	0.99 (0.80–1.22)	1.13 (0.96–1.33)	0.95 (0.81–1.11)
Polycystic ovaries or ovarian cysts	**0.95 (0.90–1.00)**	**0.89 (0.85–0.93)**	0.95 (0.89–1.01)	1.00 (0.93–1.06)	**1.05 (1.01–1.09)**	**1.04 (1.00–1.09)**
Preeclampsia^a^	**0.69 (0.63–0.75)**	**0.48 (0.44–0.52)**	**1.12 (1.00–1.25)**	**1.15 (1.04–1.28)**	1.02 (0.95–1.09)	**1.09 (1.01–1.17)**
Diabetes	**0.68 (0.53–0.85)**	**0.74 (0.60–0.92)**	1.05 (0.86–1.28)	1.01 (0.84–1.22)	1.00 (0.85–1.17)	**1.23 (1.07–1.43)**

BMI: body mass index.

^a^Includes women who had at least 1 full-term pregnancy (*n* = 58,936).

Fair agreement (0.23) was observed for the number of conditions between self-reported and NPR data. Similar with the more common conditions, hypertension and hyperlipidemia, the associations of older age, higher BMI and lower education were associated with higher agreement, using multinomial modelling for the number of conditions (data not shown).

## Discussion

Misclassification of conditions may result in confounded studies. In the study of survival, the misclassification of comorbid conditions that are associated with the higher risk of death would lead to over-emphasis of the risk of death from the disease of interest. In addition, studies looking at treatment outcomes may be potentially confounded by comorbidities. To systematically examine the appropriateness of using self-reported and register-based hospital medical (NPR) data to identify comorbidities, we compared prevalence and agreement between these two data sources in a large population-based breast cancer cohort in Sweden. Both data sources have their respective strengths and shortcomings. However, the focus of this study is not on whether self-reported personal medical history is “more correct” than NPR records and vice versa, but rather how closely they agree or disagree for various medical conditions.

Few studies have looked at the concordance of preeclampsia between self-reported and hospital data^26,27^ and to the best of our knowledge, ours is the first study investigating the same for polycystic ovaries and ovarian cysts. The Swedish NPR has been used to identify women with preeclampsia and polycystic ovaries and ovarian cysts in epidemiological studies previously^28,29^. Self-reported data provided more cases of polycystic ovaries or ovarian cysts than the NPR did and there was fair agreement between the sources. The combined classification of polycystic ovarian syndrome and ovarian cysts may have resulted in the higher than expected self-reported occurrence in the older age group. However, the prevalence estimates for preeclampsia were found to be similar for both self-reported and NPR data with moderate amount of agreement. The use of self-reported pre-eclampsia in the older generations of women may be of concern as preeclampsia may have been referred to as “toxemia of pregnancy” in the earlier period. However, in our population we observed similar concordance across the three age groups. These conditions could be potential risk factors or confounders for breast cancer risk, for example, preeclampsia has been shown to be associated with reduced risk of breast cancer^30^. Therefore, in order to identify women with these conditions reliably, both self-reported and NPR (hospital) data should be explored, if available.

Our study showed that the prevalence of hypertension and hyperlipidemia were highly under-represented in the NPR data when comparing it to self-reported data due to the fact that primary care outpatient records are not included in the NPR data. This is in agreement with previous studies which showed that medical conditions typically treated in primary care settings (not often leading to hospital admission) are not recognised or recorded in the hospital admission data. For example, a concordance study of self-reported and administrative hospital data in Australian Longitudinal Study on Women’s Health showed under-recording of hypertension in the hospital data^12^.

For life-threatening conditions like heart failure, myocardial infraction and stroke, the differences in prevalence from self-reported and NPR data were minimal. In contrast to all the comorbidities we have studied in this paper, angina was less reported in the self-reported data, for example, absolute difference in prevalence was ~10%. This might be due to the fact that angina is not a well-defined disease and many people misclassify it because its symptoms are similar to other disease (e.g. myocardial infarction) and it is perceived as a symptom, not a disease^31^. Subsequently, the agreement between self-reported angina and NPR recorded angina was poor in our study.

In spite of heterogeneous methodology and comparisons, we observed common findings among previously published studies – higher agreement for medical conditions that are widely recognized and easily diagnosed (e.g. diabetes, hypertension) or require hospital care (e.g. myocardial infarction, stroke), and lower agreement for poorly defined diseases (e.g. heart failure, angina), conditions perceived as symptoms (e.g. angina) and conditions that may not require hospitalization (e.g. hyperlipidemia). Okura *et al*. measured the agreement between self-reported cardiovascular disease and extensive medical records with high completeness (including hospital inpatient or outpatient care, office visits, emergency room and nursing home care and death certificate and autopsy information) and long archival period for ~2,000 participants from the Olmsted County in Minnesota and found substantial agreement for diabetes, hypertension, myocardial infarction and stroke (Kappa values ranging from 0.71 to 0.80)^15^. Moderate agreement was observed for heart failure (Kappa 0.46)^15^. Hamood *et al*. assessed agreement for self-reported medical history and electronic medical records (including primary and hospital care) for 119 breast cancer patients and found almost perfect agreement for diabetes (Kappa 0.93), moderate agreement for stroke (Kappa 0.79), hypertension (Kappa 0.55) and hyperlipidemia (Kappa 0.46)^18^. Agreement between self-reported and primary care data presented by Hansen *et al*. based on the MultiCare Cohort Study (*n* = 3,189) was found to be substantial for diabetes (Kappa 0.80), moderate for hypertension (Kappa 0.56) and stroke (Kappa 0.55) and fair for hyperlipidemia (Kappa 0.36)^32^. Huerta *et al*. compared self-reported diabetes, hypertension and hyperlipidemia with biometric data (levels of blood glucose and lipids and blood pressure) and found substantial (Kappa 0.78), moderate (Kappa 0.51) and fair agreement (Kappa 0.27) for the three conditions, respectively. Nonetheless, as low prevalence may result in high chance agreement, and consequently, low Kappa, caution should be exercised when interpreting statistics for less common conditions.

Overall agreement is a common measure of agreement between self-reported and hospital data^15,33^. Based on overall agreement, self-reported and NPR were concordant for 86.6% or more of the participants for all nine comorbidities studied. The high overall agreement observed in our study is mainly driven by the high negative specific agreement (>92%) for all comorbidities studied. In addition, conditions with higher proportion of positive specific agreement had higher Kappa. This may be an indication that we might be limited in identifying comorbidities when we use only one source of information. Factors associated with overall agreement tend to have similar association with positive specific agreement (Supplementary Table 7).

Previously, Ye *et al*. argued that the number of comorbidities increases with age, leading to lower precision between self-reported and medical records^33^. We observed fair agreement between self-reported and NPR data. However, we observed lower overall agreement with increased age for polycystic ovaries or ovarian cysts and preeclampsia after accounting for other factors such as education, BMI, smoking and breast cancer history, better overall agreement was observed for hypertension, hyperlipidemia and angina. Our results suggest that relationship between overall agreement and age is likely due to the length of time between disease diagnosis and study entry, as polycystic ovaries or ovarian cysts and preeclampsia are typically diagnosed at much younger ages than hypertension, hyperlipidemia or angina.

Similar to the work of Ye *et al*.^33^, we did not find education level to be a predictor of overall agreement in general, with preeclampsia being the only exception. This finding should be interpreted in the light of education in Sweden being mandatory for all children between ages 7 to age 16. In addition, higher education is available at no cost for Swedish citizens. It is unclear why better overall agreement was observed for preeclampsia. Nonetheless, women with higher education may be privileged with higher health literacy, which in turn puts them in a better position to understand information conveyed to them by physicians.

Short *et al*. hypothesized that higher BMI is correlated with lower agreement between self-reported values of healthcare utilization and administrative claims^14^. It was suggested that there might be a tendency for people with higher BMI to use more healthcare services, making it less likely for them to accurately recall and report doctor visits and inpatient hospital admissions^14^. However, in our study, self-reported diagnoses for several diseases were more likely to be confirmed by NPR data in women with higher BMI. A possible explanation may be related to the high education among women in general and also greater health consciousness of women enrolled in KARMA; they may have been more aware of the risk of chronic diseases associated with obesity. Other studies have also shown that better health status is associated with better agreement^14^. This is supported by the higher agreement observed for (polycystic ovaries or ovarian cysts, preeclampsia, and diabetes) non-smokers in our results.

The main strengths of our study include a women-only cohort, the large sample size and resulting statistical power. An electronic linkage with NPR provided complete follow-up for virtually every woman in the cohort. Although our study base comprises of women attending screening or clinical mammography, the publicly funded health care system in Sweden means that all residents have access to health care and socioeconomic bias in hospital admission is very unlikely. Nonetheless, a number of limitations warrant discussion. For example, register-based diagnoses can be complemented by information from the Swedish Drug Prescription Register (e.g. beta blockers to indicate hypertension and lipid lowering agents to indicate hyperlipidemia)^34^. However, while the Swedish Drug Prescription Register contains information regarding drug utilization and expenditures for dispensed prescribed drugs in the entire Swedish population, it was established fairly recently in July 2005 (i.e. too young to be used)^34^. There are other conditions that may be of interest, however we were limited to those in the KARMA questionnaire. Our study consisted of a highly educated population that is well-served by a mainly government-funded and decentralized health care system. It is unclear whether the results can be generalized to other populations with different health-seeking behaviour and access to healthcare. In addition, two inherent disadvantages with agreement measures must be taken into account when interpreting the results. Firstly, we acknowledge that there is no clear reference standard for the ascertainment of the medical conditions. Secondly, when the disease prevalence in the population is very high or low, the value of Cohen’s Kappa may indicate poor reliability even with a high observed proportion of agreement^35–37^ (i.e. agreement results are dependent on the disease prevalence in the study population). We have thus reported multiple measures of agreement to take into account bias, prevalence and possible imbalance in each 2 × 2 table’s marginal totals to address this paradox of the Kappa statistic.

## Conclusions

An increasing number of breast cancer cohort studies^38,39^ are including self-reported comorbidities in the data collection forms, prompting an investigation into how well the data from self-reported questionnaires correspond to register-based hospital medical data such as the Swedish NPR. Our study confirmed that on comorbidities of stroke and myocardial infarction, there is substantial overall agreement between registry data and self-reported data, regardless of age, education, and BMI. Older age was associated with better overall agreement on comorbidities of hypertension, hyperlipidemia and angina, but poorer overall agreement for polycystic ovaries or ovarian cysts, and preeclampsia. In most subgroups, negative specific agreement between registry data and self-reported data is >90%, which suggests that both sources can confidently identify individuals without the conditions studied in this subgroups.

## Supplementary information

Supplementary materials

## Data Availability

The datasets used and/or analysed during the current study are available through an application for Karma Data Access (https://karmastudy.org/data-access/) on reasonable request.
