# Supplementary material for: Comparison of self-reported and register-based hospital medical data on comorbidities in women
Source: Sci Rep. 2019 Mar 5;9:3527. doi: 10.1038/s41598-019-40072-0 (PMC6400937; doi:10.1038/s41598-019-40072-0)
Supplement: Supplementary file 1 — Supplementary materials [file 41598_2019_40072_MOESM1_ESM.pdf]

Comparison of self-reported and register-based hospital medical data on comorbidities in women

Running title: Comparing self-reported and register-based data

Peh Joo Ho[1,2]	ho_peh_joo@gis.a-star.edu.sg
Chuen Seng Tan[2]	ephtcs@nus.edu.sg
Shajedur Rahman Shawon[3]	md.shawon@dph.ox.ac.uk
Mikael Eriksson[4]	mikael.eriksson@ki.se
Li Yan Lim[5]	li_yan_lim@nuhs.edu.sg
Hui Miao[1]	miao_hui@gis.a-star.edu.sg
Eileen Png[1]	pnge@gis.a-star.edu.sg
Kee Seng Chia[2]	kee_seng_chia@nus.edu.sg
Mikael Hartman[6]	ephbamh@nus.edu.sg
Jonas F Ludvigsson[4,7,8]	jonas.ludvigsson@ki.se
Kamila Czene[4]	kamila.czene@ki.se
Per Hall[4,9]	per.hall@ki.se
*Jingmei Li[1,4,5]	lijm1@gis.a-star.edu.sg

[1] Genome Institute of Singapore, 60 Biopolis Street, Genome, #02-01, Singapore 138672, Singapore

[2] Saw Swee Hock School of Public Health, National University of Singapore and National University Health System, Singapore

[3] Cancer Epidemiology Unit, Nuffield Department of Population Health, University of Oxford, Old road campus, OX3 7LF, Oxford, UK

[4] Karolinska Institutet, Department of Medical Epidemiology and Biostatistics, Box 281, 171 77 Stockholm, Sweden

[5] Department of Surgery, University Surgical Cluster, National University Hospital, Singapore

[6] Department of Surgery, Yong Loo Lin School of Medicine, National University of Singapore, Singapore

[7] Department Medical Epidemiology and Biostatistics, Karolinska Institutet, Stockholm, Sweden

[8] Department of Pediatrics, Örebro University Hospital, Örebro University, Örebro, Sweden

[9] Department of Oncology, Södersjukhuset, 118 84 Stockholm, Sweden

***Correspondence to:** Jingmei Li, Genome Institute of Singapore, 60 Biopolis Street, Genome, #02-01, Singapore 138672, Singapore. Tel: (65) 6808 8312; Email: lijm1@gis.a-star.edu.sg

Conflict of interest: The authors declare no potential conflicts of interest.

Content

	Page
**Supplementary Method:** To show that the positive specific agreement is the inverse transformed mean of the sensitivity and positive predictive values	3
**Supplementary Table 1**. International Classification of Diseases (ICD) codes used for classifying nine medical conditions.	4
**Supplementary Table 2**. Agreement between self-reported medical conditions and hospital-documented disease in 64,961 women attending mammography units in the KARMA study	5
**Supplementary Table 3.** Subset analysis by age categories	6
**Supplementary Table 4.** Subset analysis by education categories	7
**Supplementary Table 5**. Subset analysis by body mass index (BMI) categories	8
**Supplementary Table 6**. Subset analysis by smoking status (Have you ever smoked regularly for more than 1 year or 100 cigarettes in total?)	9
**Supplementary Table 7**. Odds ratio and corresponding 95% confidence intervals for positive specific agreement in each medical condition. Significant associations (P<0.05) are denoted in bold.	10

Supplementary Method

To show that the positive specific agreement is the inverse transformed mean of the sensitivity and positive predictive values.

		Test 1	
		**Yes**	**No**
**Test 2**	**Yes**	y/y	y/n
	**No**	n/y	n/n

Sensitivity = $\frac{[y/y]}{[y/y] + [n/y]}$

Positive predictive value (PPV) = $\frac{[y/y]}{[y/y] + [y/n]}$

$$\left(\frac{1}{positive\ specific\ agreement}\right) = \frac{1}{2}\left(\frac{1}{Sensitivity} + \frac{1}{PPV}\right)$$
$$= \frac{1}{2}\left(\frac{[y/y] + [n/y]}{[y/y]} + \frac{[y/y] + [y/n]}{[y/y]}\right)$$
$$= \left(\frac{[y/y] + [n/y] + [y/y] + [y/n] + [n/n] - [n/n]}{2[y/y]}\right)$$
$$= \left(\frac{N + [y/y] - [n/n]}{2[y/y]}\right)$$

Thus, positive specific agreement = $\left(\frac{2[y/y]}{N + [y/y] - [n/n]}\right)$

Supplementary Table 1. International Classification of Diseases (ICD) codes used for classifying nine medical conditions.

	Reference	ICD7 Prior to 1968	ICD8 1969 to 1986	ICD9 1987 to 1996	ICD10 1997 to present
Hypertension	[40]	440, 441	400, 401	401	I10
Hyperlipidemia	[41]	Not available	272.4	272.4	E785
Heart failure	[42]	782.4, 434.1, 434.2	428	428	I50
Myocardial infarction	[43]		410	410	I21, I22
Angina	[44,45]	420.2	411, 413, 786	411, 413, 786	I20, I24, R07
Stroke	[46-49]	330-334	430-436	430-436	I60-64, I67-69
Polycystic ovaries or ovarian cysts	[50]	275.2	256.4, 256.9, 220.1, 615.2	256E, 620.2	E282, N832
Preeclampsia	[51]	642.2	637	642E-F	O14
Diabetes (Type I and II)	[52]	260	250	250	E10-E11

Supplementary Table 2. Agreement between self-reported medical conditions and hospital-documented disease in 64,961 women attending mammography units in the KARMA study

Condition	Self-reported No/ NPR No (*n*, %)	Self-reported Yes/ NPR No (*n*, %)	Self-reported No/ NPR Yes (*n*, %)	Self-reported Yes/ NPR Yes (*n*, %)	Percent agreement				Prevalence index	Bias index	PABAK	Cohen's Kappa	Judgement
					Overall	Expected	Positive	Negative					
Hypertension	51,380 (79.1)	7,999 (12.3)	701 (1.1)	4,881 (7.5)	86.6	75.0	52.9	92.2	0.72	0.11	0.73	0.46	Moderate
Hyperlipidemia	57,731 (88.9)	6,411 (9.9)	197 (0.3)	622 (1.0)	89.8	88.2	15.8	94.6	0.88	0.10	0.80	0.14	Slight
Heart failure	64,433 (99.2)	241 (0.4)	155 (0.2)	132 (0.2)	99.4	99.0	40.0	99.7	0.99	0	0.99	0.40	Fair
Myocardial infarction	64,391 (99.1)	144 (0.2)	88 (0.1)	338 (0.5)	99.6	98.6	74.4	99.8	0.99	0	0.99	0.74	Substantial
Angina	57,230 (88.1)	120 (0.2)	7,146 (11.0)	465 (0.7)	88.8	87.6	11.3	94.0	0.87	-0.11	0.78	0.10	Slight
Stroke	63,848 (98.3)	171 (0.3)	417 (0.6)	525 (0.8)	99.1	97.5	64.1	99.5	0.97	0	0.98	0.64	Substantial
Polycystic ovaries or ovarian cysts	57,152 (88.0)	4,492 (6.9)	1,829 (2.8)	1,488 (2.3)	90.3	86.6	32.0	94.8	0.86	0.04	0.81	0.27	Fair
Preeclampsia [1]	54,272 (92.1)	1,208 (2.0)	1,734 (2.9)	1,722 (2.9)	95.0	89.7	53.9	97.4	0.89	-0.01	0.90	0.51	Moderate
Diabetes	62,980 (97.0)	601 (0.9)	262 (0.4)	1,118 (1.7)	98.7	95.3	72.2	99.3	0.95	0.01	0.97	0.71	Substantial

Abbreviations: NPR: National Patient Register; PABAK: Prevalence and bias corrected kappa statistic
[1] Includes women who had at least 1 full-term pregnancy (*n*=58,936)

Supplementary Table 3. Subset analysis by age categories

Condition	Self-reported No/ NPR No (*n*, %)	Self-reported Yes/ NPR No (*n*, %)	Self-reported No/ NPR Yes (*n*, %)	Self-reported Yes/ NPR Yes (*n*, %)	Percent agreement				Prevalence index	Bias index	PABAK	Cohen's Kappa
					Overall	Expected	Positive	Negative				
Age<50 (n=23,154)												
Hypertension	21,422 (92.5)	1,292 (5.6)	78 (0.3)	362 (1.6)	94.1	91.2	34.6	96.9	0.91	0.05	0.88	0.33
Hyperlipidemia	22,474 (97.1)	653 (2.8)	11 (0.0)	16 (0.1)	97.1	97.0	4.60	98.5	0.97	0.03	0.94	0.04
Heart failure	23,117 (99.8)	18 (0.1)	12 (0.1)	7 (0.0)	99.9	99.8	31.8	99.9	1	0	1	0.32
Myocardial infarction	23,116 (99.8)	6 (0.0)	10 (0.0)	22 (0.1)	99.9	99.7	73.3	100.0	1	0	1	0.73
Angina	21,676 (93.6)	9 (0.0)	1,455 (6.3)	14 (0.1)	93.7	93.6	1.90	96.7	0.94	-0.06	0.87	0.02
Stroke	23,028 (99.5)	16 (0.1)	53 (0.2)	57 (0.2)	99.7	99.2	62.3	99.9	0.99	0	0.99	0.62
Polycystic ovaries or ovarian cysts	20,534 (88.7)	1,385 (6.0)	669 (2.9)	566 (2.4)	91.1	87.1	35.5	95.2	0.86	0.03	0.82	0.31
Preeclampsia [1]	58,090 (92.6)	1,208 (1.9)	1,741 (2.8)	1,723 (2.7)	95.3	90.3	53.9	97.5	0.90	-0.01	0.91	0.51
Diabetes	22,863 (98.7)	72 (0.3)	26 (0.1)	193 (0.8)	99.6	97.9	79.8	99.8	0.98	0	0.99	0.80
Age 50-59 (n=18,797)												
Hypertension	15,270 (81.2)	2,318 (12.3)	134 (0.7)	1,075 (5.7)	87.0	77.8	46.7	92.6	0.76	0.12	0.74	0.41
Hyperlipidemia	17,137 (91.2)	1,519 (8.1)	38 (0.2)	103 (0.5)	91.7	90.8	11.7	95.7	0.91	0.08	0.83	0.10
Heart failure	18,703 (99.5)	41 (0.2)	30 (0.2)	23 (0.1)	99.6	99.4	39.3	99.8	0.99	0	0.99	0.39
Myocardial infarction	18,698 (99.5)	27 (0.1)	13 (0.1)	59 (0.3)	99.8	99.2	74.7	99.9	0.99	0	1	0.75
Angina	16,720 (89.0)	14 (0.1)	1,990 (10.6)	73 (0.4)	89.3	88.7	6.8	94.3	0.89	-0.11	0.79	0.06
Stroke	18,584 (98.9)	31 (0.2)	80 (0.4)	102 (0.5)	99.4	98.3	64.8	99.7	0.98	0	0.99	0.64
Polycystic ovaries or ovarian cysts	16,386 (87.2)	1,301 (6.9)	642 (3.4)	468 (2.5)	89.7	85.8	32.5	94.4	0.85	0.04	0.79	0.27
Preeclampsia [2]	58,503 (92.6)	1,209 (1.9)	1,740 (2.8)	1,724 (2.7)	95.3	90.4	53.9	97.5	0.90	-0.01	0.91	0.51
Diabetes	18,340 (97.6)	155 (0.8)	60 (0.3)	242 (1.3)	98.9	96.3	69.2	99.4	0.96	0.01	0.98	0.69
Age≥60 (n=23,010)												
Hypertension	14,688 (63.8)	4,389 (19.1)	489 (2.1)	3,444 (15.0)	78.8	60.5	58.5	85.8	0.49	0.17	0.58	0.46
Hyperlipidemia	18,120 (78.7)	4,239 (18.4)	148 (0.6)	503 (2.2)	80.9	77.7	18.7	89.2	0.77	0.18	0.62	0.14
Heart failure	22,613 (98.3)	182 (0.8)	113 (0.5)	102 (0.4)	98.7	97.9	40.9	99.4	0.98	0	0.97	0.40
Myocardial infarction	22,577 (98.1)	111 (0.5)	65 (0.3)	257 (1.1)	99.2	97.0	74.5	99.6	0.97	0	0.98	0.74
Angina	18,834 (81.9)	97 (0.4)	3,701 (16.1)	378 (1.6)	83.5	80.9	16.6	90.8	0.80	-0.16	0.67	0.13
Stroke	22,236 (96.6)	124 (0.5)	284 (1.2)	366 (1.6)	98.2	95.2	64.2	99.1	0.95	-0.01	0.96	0.63
Polycystic ovaries or ovarian cysts	20,232 (87.9)	1,806 (7.8)	518 (2.3)	454 (2.0)	89.9	86.8	28.1	94.6	0.86	0.06	0.80	0.24
Preeclampsia [3]	58,243 (92.6)	1,209 (1.9)	1,743 (2.8)	1,725 (2.7)	95.3	90.3	53.9	97.5	0.90	-0.01	0.91	0.51
Diabetes	21,777 (94.6)	374 (1.6)	176 (0.8)	683 (3.0)	97.6	92.0	71.3	98.8	0.92	0.01	0.95	0.70

[1] Includes women who had at least 1 full-term pregnancy (*n*=20,955)
[2] Includes women who had at least 1 full-term pregnancy (*n*=17,012)
[3] Includes women who had at least 1 full-term pregnancy (*n*=20,969)
Abbreviations: NPR: National Patient Register; PABAK: Prevalence and bias corrected kappa statistic

Supplementary Table 4. Subset analysis by education categories

Condition	Self-reported No/ NPR No (*n*, %)	Self-reported Yes/ NPR No (*n*, %)	Self-reported No/ NPR Yes (*n*, %)	Self-reported Yes/ NPR Yes (*n*, %)	Percent agreement				Prevalence index	Bias index	PABAK	Cohen's Kappa
					Overall	Expected	Positive	Negative				
Elementary (n=7,793)												
Hypertension	5,019 (64.4)	1,478 (19.0)	173 (2.2)	1,123 (14.4)	78.8	61.1	57.6	85.9	0.50	0.17	0.58	0.46
Hyperlipidemia	6,240 (80.1)	1,343 (17.2)	45 (0.6)	165 (2.1)	82.2	79.0	19.2	90.0	0.78	0.17	0.64	0.15
Heart failure	7,661 (98.3)	59 (0.8)	35 (0.4)	38 (0.5)	98.8	97.8	44.7	99.4	0.98	0	0.98	0.44
Myocardial infarction	7,646 (98.1)	41 (0.5)	26 (0.3)	80 (1.0)	99.1	97.1	70.5	99.6	0.97	0	0.98	0.70
Angina	6,422 (82.4)	36 (0.5)	1,205 (15.5)	130 (1.7)	84.1	81.5	17.3	91.2	0.81	-0.15	0.68	0.14
Stroke	7,541 (96.8)	42 (0.5)	88 (1.1)	122 (1.6)	98.3	95.3	65.2	99.1	0.95	-0.01	0.97	0.64
Polycystic ovaries or ovarian cysts	6,778 (87.0)	633 (8.1)	197 (2.5)	185 (2.4)	89.3	85.6	30.8	94.2	0.85	0.06	0.79	0.26
Preeclampsia [1]	59,751 (92.7)	1,209 (1.9)	1,745 (2.7)	1,725 (2.7)	95.4	90.6	53.9	97.6	0.90	-0.01	0.91	0.51
Diabetes	7,346 (94.3)	147 (1.9)	57 (0.7)	243 (3.1)	97.4	91.5	70.4	98.6	0.91	0.01	0.95	0.69
Intermediate (n=17,721)												
Hypertension	14,270 (80.5)	2,141 (12.1)	158 (0.9)	1,152 (6.5)	87.0	76.8	50.1	92.5	0.74	0.11	0.74	0.44
Hyperlipidemia	15,991 (90.2)	1,549 (8.7)	53 (0.3)	128 (0.7)	91.0	89.7	13.8	95.2	0.90	0.08	0.82	0.12
Heart failure	17,603 (99.3)	47 (0.3)	38 (0.2)	33 (0.2)	99.5	99.2	43.7	99.8	0.99	0	0.99	0.43
Myocardial infarction	17,588 (99.2)	31 (0.2)	20 (0.1)	82 (0.5)	99.7	98.8	76.3	99.9	0.99	0	0.99	0.76
Angina	15,728 (88.8)	28 (0.2)	1,867 (10.5)	98 (0.6)	89.3	88.4	9.4	94.3	0.88	-0.10	0.79	0.08
Stroke	17,474 (98.6)	33 (0.2)	108 (0.6)	106 (0.6)	99.2	98.0	60.1	99.6	0.98	0	0.98	0.60
Polycystic ovaries or ovarian cysts	15,512 (87.5)	1,259 (7.1)	548 (3.1)	402 (2.3)	89.8	86.3	30.8	94.5	0.85	0.04	0.80	0.26
Preeclampsia [2]	58,796 (92.6)	1,209 (1.9)	1,744 (2.7)	1,724 (2.7)	95.3	90.4	53.9	97.6	0.90	-0.01	0.91	0.51
Diabetes	17,211 (97.1)	156 (0.9)	60 (0.3)	294 (1.7)	98.8	95.6	73.1	99.4	0.95	0.01	0.98	0.73
University (n=29,400)												
Hypertension	24,742 (84.2)	2,873 (9.8)	206 (0.7)	1,579 (5.4)	89.5	80.6	50.6	94.1	0.79	0.09	0.79	0.46
Hyperlipidemia	26,937 (91.6)	2,215 (7.5)	62 (0.2)	186 (0.6)	92.3	91.1	14.0	95.9	0.91	0.07	0.85	0.13
Heart failure	29,234 (99.4)	75 (0.3)	53 (0.2)	38 (0.1)	99.6	99.3	37.3	99.8	0.99	0	0.99	0.37
Myocardial infarction	29,245 (99.5)	37 (0.1)	24 (0.1)	94 (0.3)	99.8	99.2	75.5	99.9	0.99	0	1	0.75
Angina	26,493 (90.1)	27 (0.1)	2,755 (9.4)	125 (0.4)	90.5	89.8	8.2	95.0	0.90	-0.09	0.81	0.07
Stroke	29,029 (98.7)	58 (0.2)	137 (0.5)	176 (0.6)	99.3	98.2	64.4	99.7	0.98	0	0.99	0.64
Polycystic ovaries or ovarian cysts	26,075 (88.7)	1,873 (6.4)	790 (2.7)	662 (2.3)	90.9	87.3	33.2	95.1	0.86	0.04	0.82	0.29
Preeclampsia [3]	57,365 (92.5)	1,209 (1.9)	1,744 (2.8)	1,723 (2.8)	95.2	90.2	53.9	97.5	0.90	-0.01	0.90	0.51
Diabetes	28,773 (97.9)	190 (0.6)	80 (0.3)	357 (1.2)	99.1	96.7	72.6	99.5	0.97	0	0.98	0.72

[1] Includes women who had at least 1 full-term pregnancy (*n*=7,262)
[2] Includes women who had at least 1 full-term pregnancy (*n*=16,233)
[3] Includes women who had at least 1 full-term pregnancy (*n*=26,480)
Abbreviations: NPR: National Patient Register; PABAK: Prevalence and bias corrected kappa statistic

Supplementary Table 5. Subset analysis by body mass index (BMI) categories

Condition	Self-reported No/ NPR No (*n*, %)	Self-reported Yes/ NPR No (*n*, %)	Self-reported No/ NPR Yes (*n*, %)	Self-reported Yes/ NPR Yes (*n*, %)	Percent agreement				Prevalence index	Bias index	PABAK	Cohen's Kappa
					Overall	Expected	Positive	Negative				
BMI<25 (n=35,700)												
Hypertension	30,700 (86.0)	3,083 (8.6)	253 (0.7)	1,664 (4.7)	90.7	82.8	49.9	94.8	0.81	0.08	0.81	0.46
Hyperlipidemia	32,742 (91.7)	2,657 (7.4)	76 (0.2)	225 (0.6)	92.3	91.2	14.1	96.0	0.91	0.07	0.85	0.13
Heart failure	35,530 (99.5)	85 (0.2)	43 (0.1)	42 (0.1)	99.6	99.4	39.6	99.8	0.99	0	0.99	0.39
Myocardial infarction	35,502 (99.4)	48 (0.1)	35 (0.1)	115 (0.3)	99.8	99.1	73.5	99.9	0.99	0	1	0.73
Angina	32,032 (89.7)	48 (0.1)	3,474 (9.7)	146 (0.4)	90.1	89.4	7.7	94.8	0.89	-0.1	0.8	0.07
Stroke	35,204 (98.6)	72 (0.2)	202 (0.6)	222 (0.6)	99.2	98.0	61.8	99.6	0.98	0	0.98	0.61
Polycystic ovaries or ovarian cysts	31,654 (88.7)	2,309 (6.5)	1,001 (2.8)	736 (2.1)	90.7	87.4	30.8	95.0	0.87	0.04	0.81	0.26
Preeclampsia [1]	57,138 (92.4)	1,208 (2.0)	1,745 (2.8)	1,725 (2.8)	95.2	90.2	53.9	97.5	0.9	-0.01	0.9	0.51
Diabetes	35,210 (98.6)	115 (0.3)	94 (0.3)	281 (0.8)	99.4	97.9	72.9	99.7	0.98	0	0.99	0.73
BMI≥25 (n=28,800)												
Hypertension	20,304 (70.5)	4,880 (16.9)	418 (1.5)	3,198 (11.1)	81.6	66.4	54.7	88.5	0.59	0.15	0.63	0.45
Hyperlipidemia	24,555 (85.3)	3,733 (13.0)	117 (0.4)	395 (1.4)	86.6	84.4	17.0	92.7	0.84	0.13	0.73	0.14
Heart failure	28,448 (98.8)	155 (0.5)	108 (0.4)	89 (0.3)	99.1	98.5	40.4	99.5	0.98	0	0.98	0.40
Myocardial infarction	28,432 (98.7)	96 (0.3)	50 (0.2)	222 (0.8)	99.5	98.0	75.3	99.7	0.98	0	0.99	0.75
Angina	24,784 (86.1)	72 (0.2)	3,626 (12.6)	318 (1.1)	87.2	85.3	14.7	93.1	0.85	-0.12	0.74	0.13
Stroke	28,192 (97.9)	99 (0.3)	208 (0.7)	301 (1.0)	98.9	96.9	66.2	99.5	0.97	0	0.98	0.66
Polycystic ovaries or ovarian cysts	25,079 (87.1)	2,163 (7.5)	818 (2.8)	740 (2.6)	89.6	85.6	33.2	94.4	0.85	0.05	0.79	0.28
Preeclampsia [2]	57,653 (92.5)	1,209 (1.9)	1,743 (2.8)	1,722 (2.8)	95.3	90.3	53.8	97.5	0.90	-0.01	0.91	0.51
Diabetes	27,322 (94.9)	483 (1.7)	163 (0.6)	832 (2.9)	97.8	92.3	72.0	98.8	0.92	0.01	0.96	0.71

[1] Includes women who had at least 1 full-term pregnancy (*n*=32,555)
[2] Includes women who had at least 1 full-term pregnancy (*n*=26,166)
Abbreviations: NPR: National Patient Register; PABAK: Prevalence and bias corrected kappa statistic

Supplementary Table 6. Subset analysis by smoking status (Have you ever smoked regularly for more than 1 year or 100 cigarettes in total?)

Condition	Self-reported No/ NPR No (*n*, %)	Self-reported Yes/ NPR No (*n*, %)	Self-reported No/ NPR Yes (*n*, %)	Self-reported Yes/ NPR Yes (*n*, %)	Percent agreement				Prevalence index	Bias index	PABAK	Cohen's Kappa
					Overall	Expected	Positive	Negative				
Smoking Yes (n=34,274)												
Hypertension	24,662 (81.2)	3,394 (11.2)	288 (0.9)	2,036 (6.7)	87.9	77.2	52.5	93.1	0.74	0.10	0.76	0.47
Hyperlipidemia	27,416 (90.2)	2,635 (8.7)	82 (0.3)	247 (0.8)	91.1	89.6	15.4	95.3	0.89	0.08	0.82	0.14
Heart failure	30,153 (99.3)	109 (0.4)	60 (0.2)	58 (0.2)	99.4	99.1	40.7	99.7	0.99	0	0.99	0.40
Myocardial infarction	30,187 (99.4)	56 (0.2)	34 (0.1)	103 (0.3)	99.7	99.0	69.6	99.9	0.99	0	0.99	0.69
Angina	27,146 (89.4)	41 (0.1)	3,008 (9.9)	185 (0.6)	90.0	88.9	10.8	94.7	0.89	-0.10	0.80	0.10
Stroke	29,954 (98.6)	66 (0.2)	164 (0.5)	196 (0.6)	99.2	98.0	63.0	99.6	0.98	0	0.98	0.63
Polycystic ovaries or ovarian cysts	27,084 (89.2)	1,824 (6.0)	802 (2.6)	670 (2.2)	91.4	87.7	33.8	95.4	0.87	0.03	0.83	0.29
Preeclampsia [1]	57,206 (92.4)	1,208 (2.0)	1,743 (2.8)	1,724 (2.8)	95.2	90.2	53.9	97.5	0.90	-0.01	0.90	0.51
Diabetes	29,555 (97.3)	220 (0.7)	102 (0.3)	503 (1.7)	98.9	95.7	75.8	99.5	0.96	0	0.98	0.75
Smoking No (n=30,380)												
Hypertension	26,458 (77.2)	4,592 (13.4)	385 (1.1)	2,839 (8.3)	85.5	73.0	53.3	91.4	0.69	0.12	0.71	0.46
Hyperlipidemia	30,023 (87.6)	3,767 (11.0)	111 (0.3)	373 (1.1)	88.7	86.8	16.1	93.9	0.87	0.11	0.77	0.14
Heart failure	33,979 (99.1)	131 (0.4)	90 (0.3)	74 (0.2)	99.4	98.9	40.1	99.7	0.99	0	0.99	0.40
Myocardial infarction	33,902 (98.9)	88 (0.3)	50 (0.1)	234 (0.7)	99.6	98.2	77.2	99.8	0.98	0	0.99	0.77
Angina	29,821 (87.0)	79 (0.2)	4,095 (11.9)	279 (0.8)	87.8	86.5	11.8	93.5	0.86	-0.12	0.76	0.10
Stroke	33,594 (98.0)	105 (0.3)	248 (0.7)	327 (1.0)	99.0	97.1	64.9	99.5	0.97	0	0.98	0.64
Polycystic ovaries or ovarian cysts	29,781 (86.9)	2,660 (7.8)	1,018 (3.0)	815 (2.4)	89.3	85.6	30.7	94.2	0.85	0.05	0.79	0.25
Preeclampsia [2]	57,535 (92.5)	1,209 (1.9)	1,745 (2.8)	1,723 (2.8)	95.3	90.2	53.8	97.5	0.90	-0.01	0.91	0.51
Diabetes	33,125 (96.6)	380 (1.1)	156 (0.5)	613 (1.8)	98.4	95.0	69.6	99.2	0.95	0.01	0.97	0.69

[1] Includes women who had at least 1 full-term pregnancy (*n*=27,300)
[2] Includes women who had at least 1 full-term pregnancy (*n*=31,525)
Abbreviations: NPR: National Patient Register; PABAK: Prevalence and bias corrected kappa statistic

Supplementary Table 7. Odds ratio and corresponding 95% confidence intervals for positive specific agreement in each medical condition. Significant associations (P<0.05) are denoted in bold.

Condition	Age 50-59 vs Age<50	Age≥60 vs Age<50	Intermediate vs Elementary	University vs Elementary	BMI≥25 vs BMI<25	Smoking No vs Smoking Yes
Unadjusted						
Hypertension	**1.15 (1.06 – 1.26)**	**1.12 (1.04 – 1.22)**	1.02 (0.97 – 1.08)	1.04 (0.98 – 1.09)	1.04 (1.00 – 1.08)	0.99 (0.95 – 1.03)
Hyperlipidemia	1.37 (0.87 – 2.35)	1.49 (0.98 – 2.51)	0.84 (0.70 – 1.01)	0.93 (0.78 – 1.10)	1.05 (0.93 – 1.20)	0.96 (0.84 – 1.09)
Heart failure	1.29 (0.57 – 3.70)	1.41 (0.72 – 3.92)	0.92 (0.59 – 1.42)	0.76 (0.48 – 1.16)	0.94 (0.67 – 1.36)	1.08 (0.77 – 1.47)
Myocardial infarction	1.05 (0.80 – 1.44)	1.02 (0.81 – 1.36)	1.11 (0.93 – 1.33)	1.09 (0.92 – 1.30)	1.06 (0.93 – 1.21)	**0.87 (0.75 – 0.99)**
Angina	**1.65 (1.04 – 2.80)**	1.51 (0.96 – 2.50)	0.99 (0.81 – 1.22)	1.09 (0.91 – 1.33)	1.14 (0.98 – 1.35)	1.09 (0.95 – 1.25)
Stroke	0.99 (0.78 – 1.25)	0.96 (0.79 – 1.17)	1.00 (0.82 – 1.21)	1.01 (0.86 – 1.19)	1.02 (0.90 – 1.16)	0.98 (0.86 – 1.11)
Polycystic ovaries or ovarian cysts	0.89 (0.79 – 1.01)	0.98 (0.87 – 1.11)	0.85 (0.72 – 1.00)	0.94 (0.80 – 1.11)	**1.16 (1.05 – 1.29)**	1.05 (0.95 – 1.16)
Preeclampsia[1]	**0.73 (0.67 – 0.79)**	**0.35 (0.31 – 0.41)**	**1.82 (1.51 – 2.22)**	**1.83 (1.54 – 2.23)**	0.95 (0.88 – 1.03)	**1.20 (1.10 – 1.30)**
Diabetes	**0.85 (0.77 – 0.95)**	**0.86 (0.79 – 0.93)**	1.04 (0.94 – 1.16)	1.02 (0.93 – 1.13)	**1.09 (1.00 – 1.18)**	**1.09 (1.02 – 1.17)**
Multivariable-adjusted, all covariates shown						
Hypertension	**1.16 (1.07 – 1.27)**	**1.14 (1.05 – 1.24)**	1.04 (0.98 – 1.09)	1.05 (0.99 – 1.10)	1.04 (1.00 – 1.08)	1.00 (0.96 – 1.03)
Hyperlipidemia	1.34 (0.86 – 2.29)	1.43 (0.94 – 2.42)	0.87 (0.72 – 1.05)	0.95 (0.80 – 1.14)	1.05 (0.92 – 1.20)	0.96 (0.84 – 1.09)
Heart failure	1.38 (0.59 – 4.20)	1.49 (0.73 – 4.36)	0.96 (0.62 – 1.55)	0.76 (0.48 – 1.17)	0.90 (0.64 – 1.33)	1.08 (0.76 – 1.50)
Myocardial infarction	1.03 (0.77 – 1.43)	1.04 (0.80 – 1.40)	1.14 (0.95 – 1.40)	1.12 (0.93 – 1.34)	1.06 (0.93 – 1.23)	0.87 (0.75 – 0.99)
Angina	**1.68 (1.06 – 2.82)**	1.53 (0.96 – 2.53)	1.01 (0.81 – 1.24)	1.11 (0.92 – 1.36)	1.16 (0.99 – 1.37)	1.10 (0.96 – 1.27)
Stroke	0.98 (0.77 – 1.24)	0.94 (0.76 – 1.18)	0.98 (0.80 – 1.20)	1.00 (0.85 – 1.19)	1.03 (0.90 – 1.16)	0.98 (0.86 – 1.11)
Polycystic ovaries or ovarian cysts	0.88 (0.78 – 1.01)	0.95 (0.83 – 1.08)	0.85 (0.71 – 1.02)	0.95 (0.81 – 1.13)	1.17 (1.06 – 1.30)	1.04 (0.94 – 1.16)
Preeclampsia[1]	**0.75 (0.69 – 0.82)**	**0.38 (0.33 – 0.43)**	**1.29 (1.06 – 1.58)**	**1.37 (1.14 – 1.68)**	1.02 (0.94 – 1.11)	1.08 (0.99 – 1.18)
Diabetes	**0.87 (0.79 – 0.97)**	**0.87 (0.81 – 0.95)**	1.02 (0.92 – 1.14)	1.00 (0.91 – 1.11)	**1.10 (1.01 – 1.19)**	**1.08 (1.01 – 1.16)**

BMI: body mass index
[1] Includes women who had at least 1 full-term pregnancy
